# Efficient estimation of generalized linear latent variable models

**DOI:** 10.1371/journal.pone.0216129

**Published:** 2019-05-01

**Authors:** Jenni Niku, Wesley Brooks, Riki Herliansyah, Francis K. C. Hui, Sara Taskinen, David I. Warton

**Affiliations:** 1 Department of Mathematics and Statistics, University of Jyväskylä, Jyväskylä, Finland; 2 School of Mathematics and Statistics, The University of New South Wales, Sydney, Australia; 3 Department of Mathematics, Kalimantan Institute of Technology, Kalimantan, Indonesia; 4 Research School of Finance, Actuarial Studies & Statistics, Australian National University, Canberra, Australia; 5 Evolution & Ecology Research Centre, The University of New South Wales, Sydney, Australia; Geoscience Australia, AUSTRALIA

## Abstract

Generalized linear latent variable models (GLLVM) are popular tools for modeling multivariate, correlated responses. Such data are often encountered, for instance, in ecological studies, where presence-absences, counts, or biomass of interacting species are collected from a set of sites. Until very recently, the main challenge in fitting GLLVMs has been the lack of computationally efficient estimation methods. For likelihood based estimation, several closed form approximations for the marginal likelihood of GLLVMs have been proposed, but their efficient implementations have been lacking in the literature. To fill this gap, we show in this paper how to obtain computationally convenient estimation algorithms based on a combination of either the Laplace approximation method or variational approximation method, and automatic optimization techniques implemented in R software. An extensive set of simulation studies is used to assess the performances of different methods, from which it is shown that the variational approximation method used in conjunction with automatic optimization offers a powerful tool for estimation.

## 1 Introduction

High-dimensional multivariate abundance data, which consist of records (e.g. species counts, presence-absence records, and biomass) of a large number of interacting species at a set of units or sites, are routinely collected in ecological studies. When analyzing multivariate abundance data, the interest is often in visualization of correlation patterns across species, hypothesis testing of environmental effects, and making predictions for abundances. Classical methods for analysing such data, including algorithmic-based approaches such as non-metric multidimensional scaling (nMDS) and correspondence analysis (CA), are based on distance matrices computed on some pre-specified dissimilarity measure [1]. As such, they often make wrong assumptions for key properties of the data at hand (e.g. mean-variance relationship), which can potentially lead to misleading inferential results [2, 3].

An alternative approach that has gained considerable attention over the past several years is generalized linear latent variable models (GLLVMs, [4]). GLLVMs start with the basic generalized linear model (GLM, [5]), classically used to model the impact of environmental covariates on abundance of one species, and extend it by incorporating latent variables to model between response correlation in a parsimonious manner. As the model makes explicit assumptions concerning the response distribution, the mean-variance relationship can be correctly modeled and verified using (for instance) residual analysis and model selection approaches. In the context of multivariate abundance data, GLLVMs were first proposed by [6] for presence-absence data, and [7] in a more general framework for model-based unconstrained ordination. By adding covariates to the model, it can be used as a model-based approach to correspondence analysis [8]. More recently, there has been an explosion in research on various extensions of GLLVMs for joint analyses of multivariate abundance data, see [9–12] among many others.

One of the main and long standing challenges with using GLLVMs is the lack of computationally efficient estimation methods. The need for fast and efficient estimation methods evolves from the fact that modern data collection tools such as metabarcoding often result in very large and high-dimensional datasets (for a recent review, see [13]), and current methods are unable to fit GLLVMs for such data in reasonable amount of time. Specifically, many of the standard methods proposed in the literature for fitting GLLVMs have a major drawback as being either computationally very intensive with high-dimensional data e.g. the Expectation Maximization algorithm [7, 14] and Bayesian Markov Chain Monte Carlo estimation [11, 15], or are computationally impractical with a larger number of latent variables, such as Gauss-Hermite quadrature [16–18]. In recent years, a number of approaches have been proposed in the literature to overcome such issues, with two of the more prominent ones being the variational approximation method to approximate the likelihood in the case of binary, ordinal and overdispersed count data [19], and the Laplace approximation method for responses from the exponential family of distributions [20], which has recently been adapted specifically for overdispersed count and biomass data in ecology [21]; Note that the Laplace approximation can be considered as a special case of adaptive quadrature with only one quadrature point. Both estimation methods provide a closed form approximation to the marginal log-likelihood that can then be maximized efficiently.

In this paper, we propose a framework for faster fitting of GLLVMs using either Laplace approximation method or the variational approximation method. Our method utilizes the R package TMB (Template Model Builder, [22]), which offers a general tool for implementing complex random effect models through simple C++ templates. TMB is inspired by AD Model Builder [23], which is a C++ language extension for solving optimization problems using automatic differentiation [24]. With growing popularity, TMB has been used to estimate complex non-linear models, e.g. for fitting mixed-effect models [25] and non-Gaussian state space models [26]. The algorithms we propose in this article for efficient estimation of GLLVMs have been recently implemented in the R package gllvm [27].

Another major contribution we make is to provide a new method for obtaining starting values for parameter estimation of GLLVMs. This is especially important for GLLVMs given their complex mean and latent variable structures may cause the observed likelihood to be multimodal (as discussed in [28]), and good starting values are therefore critical in order to guarantee fast convergence and to avoid local maxima. Our proposed method is based around fitting univariate GLMs to each species in order to obtain starting values for fixed parameters, and then applying a factor analysis to the Dunn-Smyth residuals [29] from the fitted GLMs as the basis for constructing starting values for the loadings and latent variables. We performed an extensive series of simulation studies to compare the performances of estimation algorithms with and without TMB, and to compare various methods for constructing starting values. The simulation studies showed that in most cases, the variational approximation method utilizing TMB outperformed the other estimation algorithms: computation times were clearly faster than those of the other methods, the empirical mean biases and mean squared errors of the parameter estimates were smaller, and coverage probabilities of Wald-type confidence intervals were closer to their nominal level. Our simulations also show that the proposed approach for choosing starting values outperformed more standard methods such as random starting values in terms of consistency of reaching the global maximum of the likelihood, regardless of the data at hand.

The paper is organized as follows. In Section 2, we formulate a generalized linear latent variable model suitable for joint modeling of abundance data, and review the most recently proposed approximation methods. In Section 3, we explain how the estimation can be performed using TMB and introduce different methods for obtaining starting values for estimation. In section 4, we study the performances of our methods using several simulation studies. Section 5 concludes the paper.

## 2 Generalized linear latent variable models

Consider a sample of observations consisting of responses for *m* species collected at *n* sites, such that *y*_*ij*_ denotes the response for species *j* = 1, …, *m* at site *i* = 1, …, *n*. A generalized linear latent variable model (GLLVM) regresses the mean response, denoted here as *μ*_*ij*_, against a vector of *d* ≪ *m* latent variables, **u**_*i*_ = (*u*_*i*1_, …, *u*_*id*_)′, along with the vector of covariates ***x***_*i*_ = (*x*_*i*1_, …, *x*_*ik*_)′. That is,

$$\begin{array}{c} g\left(\mu_{ij}\right)=\eta_{ij}=\alpha_{i}+\beta_{0j}+x_{i}^{\prime }\beta_{j}+u_{i}^{\prime }\gamma_{j}, \end{array}$$ (1)

where ***β***_*j*_ and ***γ***_*j*_ are vectors of species specific coefficients related to the covariates and latent variables, respectively. It is the term $u_{i}^{\prime }\gamma_{j}$ which captures the residual correlation across species not accounted for by the observed covariates *x*_*i*_. Moreover, a key advantage of this type of model is that it is capable of flexibly handling correlation across response variables in a parsimonious manner, with the number of parameters characterizing the correlation structure growing linearly in the number of responses *m*. This allows GLLVMs to be feasibly fitted to datasets with relatively large *m*, as often arises in practice [8].

We assume that the latent variables follow a multivariate standard normal distribution, ***u***_*i*_ ∼ *N*_*d*_(**0**, ***I***_*d*_), where ***I***_*d*_ denotes a *d* × *d* identity matrix. The assumption of zero mean and unit variance is made in order to fix the locations and scales of latent variables. We also set all the upper triangular elements of *m* × *d* matrix Γ = (***γ***_1_⋯***γ***_*m*_)′ to be zero, that is, *γ*_*ij*_ = 0 for *j* > *i*, and constrain its diagonal elements, *γ*_*ii*_, to be positive in order to avoid rotation invariance and to ensure parameter identifiability.

For the GLLVM defined in Eq (1), where the *α*_*i*_’s are assumed to be random row effects (reflecting a nested sampling design, say), denote $u_{i}^{*}={\left(\alpha_{i},u_{i}^{\prime }\right)}^{\prime }$ and $\gamma_{j}^{*}={\left(1,\gamma_{j}^{\prime }\right)}^{\prime }$ and write the model as $g\left(\mu_{ij}\right)=\eta_{ij}=\beta_{0j}+x_{i}^{\prime }\beta_{j}+{u_{i}^{*}}^{\prime }\gamma_{j}^{*}$. Since the latent variables and random intercepts are assumed to be independent, then $u_{i}^{*}$ follows a multivariate normal distribution with mean zero and block diagonal covariance matrix, ***C***_*σ*^2^_ = bdiag(*σ*^2^, ***I***_*d*_), where bdiag(⋅) is the block diagonal operator. Write the probability density function of *N*(**0**, ***C*_*σ*^2^_**) as $f(u_{i}^{*};\sigma^{2})$. To complete the formulation, we assume that conditional on the latent variables $u_{i}^{*}$ and parameter vector **Ψ**, the responses are independent observations from the exponential family of distributions with probability density function,

$$\begin{array}{c} f\left(y_{ij}|u_{i},\Psi \right)=\exp\{\frac{y_{ij}a\left(\eta_{ij}\right)-b\left(\eta_{ij}\right)}{\phi_{j}}+c\left(y_{ij};\phi_{j}\right)\}, \end{array}$$ (2)

where *a*(⋅), *b*(⋅) and *c*(⋅) are known functions and *ϕ*_*j*_ is a species specific dispersion parameter. Let $\Psi =(\beta_{0}^{\prime },vec{\left(B\right)}^{\prime },vec{\left(\Gamma \right)}^{\prime },\Phi^{\prime },\sigma^{2})$ denote the full vector of parameters in the GLLVM, where ***β***_0_ = (*β*_01_, …, *β*_0*m*_)′, ***B*** = (*β*_1_…*β*_*m*_)′, Γ = (*γ*_1_…*γ*_*m*_)′, and Φ includes all other nuisance parameters e.g. Φ = (*ϕ*_1_, …, *ϕ*_*m*_)′. With the conditional independence of the responses given the latent variables, we then obtain $f\left(y_{i},u_{i}^{*},\Psi \right)=\prod_{j=1}^{m}f\left(y_{ij}|u_{i}^{*};\Psi \right)f\left(u_{i}^{*};\sigma^{2}\right)$ as the joint distribution. By integrating over latent variables $u_{i}^{*}$ then, we obtain the following marginal log-likelihood function for a GLLVM,

$$\begin{array}{c} l\left(\Psi \right)=\sum_{i=1}^{n}\log\left(f\left(y_{ij},\Psi \right)\right)=\sum_{i=1}^{n}\log(\int_{R^{d+1}}\prod_{j=1}^{m}f\left(y_{ij}|u_{i}^{*};\Psi \right)f\left(u_{i}^{*};\sigma^{2}\right)du_{i}^{*}). \end{array}$$ (3)

For non-normal responses the above log-likelihood cannot be solved analytically. To overcome the integral in Eq (3), we consider in the following section closed-form approximations for the likelihood.

### 2.1 Approximations to the marginal likelihood of GLLVMs

Computationally, the most efficient likelihood based approaches for estimating GLLVMs are methods which approximate the marginal likelihood in a closed form. Of these, the most common and well known is the Laplace approximation method, which has been used extensively in the statistical literature to approximate marginal likelihood functions that cannot be resolved analytically [30]. The Laplace approximation can be easily applied to a marginal likelihood $l(\Psi )=\sum_{i=1}^{n}\text{log}\int f(y_{i}|u_{i}^{*},\Psi )f(u_{i}^{*})\;du_{i}^{*}$ with latent variables $u_{i}^{*}$. By denoting $Q(y_{i},u_{i}^{*},\Psi )=\text{log}\{f(y_{i}|u_{i}^{*},\Psi )f(u_{i}^{*})\}/m$, the likelihood can be written as $l(\Psi )=\sum_{i=1}^{n}\text{log}\;\int \text{exp}(mQ(y_{i},u_{i}^{*},\Psi ))\;du_{i}^{*}$. Assuming further that ${\hat{u}}_{i}^{*}$ maximizes $Q(y_{i},u_{i}^{*},\Psi )$, the Laplace approximation method applies a second order Taylor expansion for $Q(y_{i},u_{i}^{*},\Psi )$ around the maximum ${\hat{u}}_{i}^{*}$, and thus allows the integral to be performed in a tractable manner (it resembles the normalization constant for a multivariate normal distribution). For GLLVMs, the Laplace approximation was first proposed in [20], and extended by [21] to handle important distributions arising in ecology such as the negative binomial, Poisson, zero inflated Poisson and Tweedie distributed responses. For a model as defined in Eq (1) with random row effects and responses *y*_*ij*_ coming from the exponential family of distributions with mean *μ*_*ij*_ as defined in (2), the Laplace approximation of the marginal log-likelihood function can be written as follows:

$$\begin{array}{cc} \tilde{l}\left(\Psi \right) & =\sum_{i=1}^{n}(-\frac{1}{2}\log\det\{G(\Psi ,{\hat{u}}_{i}^{*})\}+\sum_{j=1}^{m}\{\frac{y_{ij}\;a\left({\hat{\eta }}_{ij}\right)-b\left({\hat{\eta }}_{ij}\right)}{\phi_{j}}+c\left(y_{ij};\phi_{j}\right)\}\\ & \;-\frac{1}{2}{{\hat{u}}_{i}^{*}}^{\prime }C_{\sigma^{2}}^{-1}{\hat{u}}_{i}^{*}-\frac{1}{2}\log\det\left(C_{\sigma^{2}}\right)), \end{array}$$

where

$$G\left(\Psi ,{\hat{u}}_{i}^{*}\right)=\sum_{j=1}^{m}\frac{\partial^{2}\{-y_{ij}\;a\left(\eta_{ij}\right)+b\left(\eta_{ij}\right)\}}{\partial {u_{i}^{*}}^{\prime }\partial u_{i}^{*}}{|}_{u_{i}^{*}={\hat{u}}_{i}^{*}}+C_{\sigma^{2}},$$

${\hat{\eta }}_{ij}=\beta_{0j}+x_{i}^{\prime }\beta_{j}+{{\hat{u}}_{i}^{*}}^{\prime }\gamma_{j}^{*}$, ***C***_*σ*^2^_ = *bdiag*(*σ*^2^, ***I***_*d*_), ${\hat{u}}_{i}^{*}={\left(\alpha_{i},u_{i}^{\prime }\right)}^{\prime }$ and ${\hat{u}}_{i}^{*}$ maximizes

$$\begin{array}{cc} Q(y_{i},u_{i}^{*},\Psi ) & =\frac{1}{m}(\sum_{j=1}^{m}\{\frac{y_{ij}a\left(\eta_{ij}\right)-b\left(\eta_{ij}\right)}{\phi_{j}}+c\left(y_{ij};\phi_{j}\right)\}-\frac{1}{2}{u_{i}^{*}}^{\prime }C_{\sigma^{2}}^{-1}u_{i}^{*}\\ & \;-\frac{1}{2}\log\det\left(C_{\sigma^{2}}\right)) \end{array}$$

with respect to $u_{i}^{*}$. All quantities that are constant with respect to the parameters have been omitted. Some further simplification of this expression is possible when the model is defined using a canonical link function [21].

When using Laplace approximations, the estimation is performed by maximizing $\tilde{l}(\Psi )$ with respect to **Ψ**, and $Q(y_{i},u_{i}^{*},\Psi )$ with respect to $u_{i}^{*}$. The estimates ${\hat{u}}_{i}^{*}$ are then used as predictions of the latent variables. Furthermore, asymptotic standard errors for $\hat{\Psi }$ and ${\hat{u}}_{i}^{*}$ are computed as the negative Hessian matrix obtained as part of the estimation process. These may form the basis for performing statistical inference for the model parameters and evaluate prediction errors for the latent variables, both of which will be examined empirically in the simulation studies in Section 4.

Another method which allows us to derive a closed form approximation for the marginal likelihood is the variational approximation method. The idea of variational approximations originates from machine learning research, where it is often used to approximate probability densities [31]. More recently, the method has gained considerable traction in Bayesian data analysis for efficiently approximating posterior densities [32, 33]. The variational approximation method is also applicable in likelihood based contexts for approximating an intractable marginal likelihood [34], although it is less frequently used in this context. Furthermore, the large sample properties of estimates and inference obtained using the variational approximation method are not thoroughly studied and remain a topic of future research [33].

The main idea behind likelihood based variational approximations is to approximate the posterior distribution of the random effects i.e., $f(u_{i}^{*}|y_{i},\Psi )$ by a simpler distribution in order to get a closed form (or almost closed-form) expression for the marginal log-likelihood. This so called variational likelihood is a strict lower bound to the marginal log-likelihood, and is then treated as the new objective function on which to base estimation and inference. In practice, for a marginal log-likelihood function $l(\Psi )=\sum_{i=1}^{n}\text{log}\int f(y_{i}|u_{i}^{*},\Psi )f(u_{i}^{*})\;du_{i}^{*}$, the variational approximation approach make use of Jensen’s inequality to construct this lower bound,

$$\begin{array}{cc} \sum_{i=1}^{n}\log\int f\left(y_{i}|u_{i}^{*},\Psi \right)f\left(u_{i}^{*}\right)du_{i}^{*} & =\sum_{i=1}^{n}\log\int \{\frac{f\left(y_{i}|u_{i}^{*},\Psi \right)f\left(u_{i}^{*}\right)q\left(u_{i}^{*}|\xi \right)}{q(u_{i}^{*}|\xi )}\}du_{i}^{*}\\ & \geq \sum_{i=1}^{n}\int \log\{\frac{f\left(y_{i}|u_{i}^{*},\Psi \right)f\left(u_{i}^{*}\right)}{q(u_{i}^{*}|\xi )}\}q\left(u_{i}^{*}|\xi \right)du_{i}^{*}, \end{array}$$

for some variational density $q(u_{i}^{*}|\xi )$ with variational parameters **ξ**. Critically, the logarithm can be brought inside the integral, thereby making integration easier for the exponential family of distributions. By maximizing the variational log-likelihood with respect to both the model parameters **Ψ** and variational parameters **ξ**, we see that maximizing the variational likelihood is equivalent to minimizing the Kullback-Leibler divergence between the true posterior, $f(u_{i}^{*}|y_{i},\Psi )$, and the proposed variational density $q(u_{i}^{*}|\xi )$.

The variational approximation method was applied to the estimation of GLLVMs by [19] and it was shown that it is optimal in some sense to choose, as variational densities *q*(⋅), independent normal distributions for the latent variables for each observational unit. Following on from this, for our GLLVM model in Eq (1) with random row effects we choose $q(u_{i}^{*}|\xi_{u_{i}^{*}})=N_{d+1}(a_{i},A_{i})$ for *i* = 1, …, *n*, where $\xi_{u_{i}^{*}}=(a_{i},\text{vec}(A_{i}{)}^{\prime }{)}^{\prime }$, $A_{i}=bdiag\left(A_{\alpha_{i}},A_{u_{i}}\right)$ and $A_{u_{i}}$ is an unstructured *d* × *d* covariance matrix. For responses coming from the exponential family of distributions with the canonical link function, this leads to the variational approximation of the GLLVM log-likelihood as follows:

$$\begin{array}{cc} \underline{\ell }\left(\Psi ,\xi \right)= & \sum_{i=1}^{n}\sum_{j=1}^{m}\{\frac{y_{ij}{\tilde{\eta }}_{ij}-E_{q^{*}}\left\{b\left(\eta_{ij}\right)\right\}}{\phi_{j}}+c\left(y_{ij},\phi_{j}\right)\}\\ & +\frac{1}{2}\sum_{i=1}^{n}(\log\det\left(A_{i}\right)-\text{tr}\left(C_{\sigma^{2}}^{-1}A_{i}\right)-a_{i}^{\prime }C_{\sigma^{2}}^{-1}a_{i}-\log\det\left(C_{\sigma^{2}}\right)), \end{array}$$

where ${\tilde{\eta }}_{ij}=\beta_{0j}+x_{i}^{\prime }\beta_{j}+a_{i}^{\prime }\gamma_{j}^{*}$, ***C***_*σ*^2^_ = *bdiag*(*σ*^2^, ***I***_*d*_) and *a*_*i*_ and ***A***_*i*_ are the mean and the covariance matrix of a variational density, respectively. All quantities constant with respect to the parameters have been omitted. Notice the lower bound includes the expectation term *E*_q*_{*b*(*η*_*ij*_)}, which is not guaranteed to have a closed form for any distribution form the exponential family. Through reparameterization of the GLLVM, fully explicit forms for $\underline{\ell }(\Psi ,\xi )$ can be derived for some common occurring responses in multivariate abundance data, such as binary, ordinal and overdispersed count responses [19].

One attractive feature of likelihood based variational approximations is that the estimated means of the variational distributions, ${\hat{a}}_{i}$, *i* = 1, …, *n*, provide a natural predictor for the latent variables $u_{i}^{*}$, while the estimated covariance matrices ${\hat{A}}_{i}$ along with the assumed variational density $q(u_{i}^{*}|\xi )$ can be used as the basis for constructing prediction intervals [34]. Both quantities are obtained directly from the maximization procedure. Furthermore, asymptotic standard errors for the model parameters can be obtained by using the block inverse matrix of the negative Hessian of $\underline{\ell }(\Psi ,\xi )$, (see also [35]).

## 3 Implementation

Two advances are made in this paper, which enable faster, more reliable fitting of GLLVMs than previous implementations of Laplace or variational approximations. First, we write software to make use of automatic differentiation software in the TMB package [22]. Secondly, we make strategic choices for the starting values of the parameters in the GLLVM, in order to improve speed and stability of the estimation algorithms. Our simulations presented later demonstrate that these changes improve speed by an order of magnitude, as well as improving reliability by increasing the accuracy of the estimates.

### 3.1 Implementation with TMB

The closed form approximate marginal log-likelihoods proposed in the previous section are often maximized using some gradient-based optimization algorithms. This presents a computational challenge as it means that the gradient functions need to be calculated for each response distribution and specific model separately. To overcome this, we use Template Model Builder (TMB) for fitting GLLVMs. TMB is a general R package for fitting non-linear mixed effects and latent variable models based on AD Model Builder, which is a C++ language extension for solving statistical optimization problems using automatic differentiation techniques [23]. To perform optimization using TMB in general, the complete log-likelihood for the model of interest is written in C++, from which TMB employs the C++ library ‘CppAD’ to efficiently construct functions for calculating the associate gradient and Hessian. These functions written can then be called from R, and can be straightforwardly passed into gradient based optimization methods such as optim() or nlminb(). After optimization, the Hessian matrix is obtained as a side product and can be used to calculate standard errors for parameters. Note however initialization of the model and the choice of starting values must be done in R.

For models involving random effects, TMB uses the Laplace approximation method. As a result, we can straightforwardly adapt it for maximizing the Laplace approximation of the GLLVM log-likelihood in Section 2.1 based on the following steps:

1. Write the complete log-likelihood for the responses and latent variables in C++ using the TMB model template and compile it.
2. Set initial values for the model parameters and the latent variables in R; see Section 3.2.
3. Create the TMB object using TMB::MakeADFun() with data, initial values and the objective function as input, specifying the names of the parameters to be integrated out of the likelihood using argument random in TMB::MakeADFun(). The Laplace approximation method will then be automatically applied to the complete likelihood, and gradient and Hessian functions for the marginal log-likelihood will be constructed.
4. Optimize the objective function using optim() or nlminb() in R.
5. Calculate the Hessian matrix in R using optimHess(), from which the standard errors for the model parameters as well as prediction errors for the latent variables can be obtained.

Notice that the initialization in Step 2 is crucial for the model fitting as poor initial values may yield to convergence problems. We return to the selection of starting values in Section 3.2.

Since TMB allows maximization of any likelihood function, it can also be used to optimize the variational approximation to the marginal log-likelihood for GLLVMs. In this case, we can treat the variational parameters **ξ** as additional model parameters and maximize the variational approximation to the log-likelihood based on the following steps:

1. Write the variational approximation lower bound for the log-likelihood in C++ using TMB model template and compile it.
2. Set initial values for the model parameters and the variational parameters in R; see Section 3.2.
3. Create the TMB object using TMB::MakeADFun() with data, initial values and the objective function as input. The gradient and Hessian for the variational approximated log-likelihood will then be automatically calculated using TMB::MakeADFun().
4. Optimize the objective function using optim() or nlminb() in R.
5. Calculate the Hessian matrix in R using optimHess(), from which standard errors for the model parameters as well as prediction errors for the latent variables may be obtained by applying block inversion for the negative Hessian matrix.

Finally, for all the implementations we considered, we parameterized any dispersion parameters and variance components in terms of their log transformed values in to avoid boundary issues in estimation and inference i.e. log(*σ*), log(*ϕ*), and so on.

### 3.2 Starting values

With GLLVMs and models involving a large number of latent random effects, the importance of selecting the initial values of model parameters is particularly important. When the observed likelihood function is multimodal, maximization algorithms can often end up in local maxima if the initial values for parameters are not sufficiently close enough to the global maximum. A widely used strategy to work around this issue is to use several random starting values and to pick up the solution with highest log-likelihood value. In case of complex models and large datasets however, the use of several random starting values may however be too time consuming.

We propose a new data driven method for constructing initial values for parameters in a GLLVM. In this approach, we first fit a GLM, $g\left(E\left(y_{ij}\right)\right)=\beta_{0j}+x_{i}^{\prime }\beta_{j}$, to each response variable (species), from which the obtained estimates of *β*_0*j*_ and *β*_*j*_ are used as starting values for the fixed parameters in the GLLVM. Starting values for latent variables *u*_*i*_ and their loadings *γ*_*j*_ are then constructed by applying factor analysis to the Dunn-Smyth residuals [29] from the fitted GLMs. Furthermore, the matrices of starting values for the latent variables and the loadings obtained via factor analysis are rotated so that the upper triangle of the loading matrix is zero, so as to adhere to the parameter identifiability constructed below Eq (1). As starting values for the random row effects, we use a vector of zeros. The key idea underlying this approach to constructing starting values lies in the Dunn-Smyth residuals, which are defined for the observation *y*_*ij*_ as

$$\begin{array}{c} r_{ij}=\Phi^{-1}\left(z_{ij}F_{ij}\left(y_{ij}\right)+\left(1-z_{ij}\right)F_{ij}^{-}\left(y_{ij}\right)\right), \end{array}$$ (6)

where Φ and *F*_*ij*_ are the cumulative distribution functions of the standard normal distibution and the response variable, respectively, $F_{ij}^{-}$ is the limit as *F*_*ij*_ is approached from the negative side, and *z*_*ij*_ is a random variable generated from the standard uniform distribution. Dunn-Smyth residuals have the attractive property that if model assumptions are correct, then the residuals are exactly normally distributed. The normality of the residuals motivates us to use the classical factor analysis on the residuals from the fitted GLMs, in particular, because they contain information regarding the residual correlation across species not accounted for by the observed covariates. For the remainder of this article, we will refer to this method for constructing starting values as res.

An extension to the above method is resX, where the starting values are obtained in a similar fashion as in res, with the crucial difference being that resX uses *X* sets of starting values for the latent variables. These are obtained by “jittering” starting values by adding random variation from a normal distribution to the latent variables obtained using res. In our simulation studies we use a jitter variance of 0.2^2^ and *X* = 3 sets of starting values (we will thus refer to this approach as res3 in Section 4). With *X* sets of starting values, which only differ in the latent variables (the starting values for the ***B***, **Γ**, and **Φ** remain the same), the estimation procedure then proceeds as we would with random starting values. That is, a GLLVM is fitted using those *X* different sets of starting values, and the fit with the highest log-likelihood value is then considered the best fitting GLLVM for that dataset.

In the simulation studies in the following section, we will compare res and res3 to two alternative and common methods for constructing starting values: 1) a method referred to as zero, where we use zero initial values for all parameters; 2) a method referred to as random, where we simulate initial values for latent variables from a multivariate standard normal distribution, while (as previously) a GLM is fitted to each response variable against environmental variables and latent variables to get starting values for fixed parameters and loadings. Note that the difference between random and res/res3 is that the latter makes use of the residual information from the multivariate GLM to directly construct the starting values for the latent variables and loadings, while the former simulates these randomly.

## 4 Simulation studies

We performed a series of simulation studies to compare the performance of different model fitting algorithms with and without automatic differentiation using TMB, using either the Laplace approximation or variational approximation, and with different starting value strategies (res, res3, zero, random). For fitting algorithms without automatic differentiation, we implemented both the Laplace and variational approximations in plain R code by manually defining their respective approximate likelihoods and their gradient functions. Details of the simulation design are given below.

### 4.1 Simulation designs

We considered GLLVMs with multivariate count and binary data, and based our simulation studies on two real datasets: the first dataset consists of abundances of testate amoebae in Finnish peatlands [36], and the second dataset consists of abundances of bird species in Indonesia [37].

The first simulation setup was based on the testate amoebae data [36], which consist of counts of *m* = 48 testate amoebae species measured from *n* = 263 sampling sites across six peatlands in southern and central Finland. Two environmental variables, water pH and water temperature, were also recorded at each sampling site. We conducted simulation studies based on the original count data as well as based on binary data obtained by converting counts to presence-absences. As mean models, we used $\log\left(\mu_{ij}\right)=\beta_{0j}+x_{i}^{\prime }\beta_{j}+u_{i}^{\prime }\gamma_{j}$ for counts and $\Phi^{-1}\left(\mu_{ij}\right)=\beta_{0j}+x_{i}^{\prime }\beta_{j}+u_{i}^{\prime }\gamma_{j}$ for presence-absences, where *x*_*i*_ includes the values for the two covariates recorded at site *i*, and *u*_*i*_ includes two latent variables. Notice that with two-dimensional latent variables, GLLVMs can be used as a model-based ordination method as described in [7]. The parameters for the true model used to simulate multivariate abundance data were obtained by fitting a negative binomial (Bernoulli) GLLVM to the real data, consisting of counts (presence-absences) of observed amoebae species. To study the effect of sample size on performance, we constructed nested subsets of size *n* = 50, 120, 190 and 260 randomly sampling from the sites and used parameters of the fitted model, which corresponded the sites in subsets, to generate datasets of the desired sizes. We generated *K* = 500 datasets for each value of *n*, and for each dataset we fitted GLLVMs using the four starting value strategies and both approximation methods with and without automatic differentiation.

The second simulation setup was based on Indonesian bird data [37], which consists of counts of *m* = 177 bird species measured from *n* = 37 sites in Central Kalimantan, Indonesia. We conducted a simulation study for the original count data as well as for the binary data obtained by converting counts to presence-absences. We used $\log\left(\mu_{ij}\right)=\beta_{0j}+u_{i}^{\prime }\gamma_{j}$ for counts and $\Phi^{-1}\left(\mu_{ij}\right)=\beta_{0j}+u_{i}^{\prime }\gamma_{j}$ for presence-absence data, with parameters for the true model based on a negative binomial GLLVM fitted to the count data and a Bernoulli GLLVM fitted to the binary data. In this simulation study, we varied the number of species, that is, we used four different numbers of randomly selected species, *m* = 30, 60, 100 and 140. As in the previous setup, the parameters for the true model were obtained by fitting a negative binomial (Bernoulli) GLLVM to the data in the case of counts (presence-absences), and the parameters that corresponded the species in each subset were used obtain a dataset of the desired size. For each value of *m*, we generated *K* = 500 datasets, and for each dataset we fitted GLLVMs using four different starting value strategies and both approximation methods with and without automatic differentiation.

In addition to the above two simulation setups, we included another design based on the Indonesian birds data, where we added a random row effect to the simulation model. Specifically, the true mean models were given by $\log\left(\mu_{ij}\right)=\alpha_{i}+\beta_{0j}+u_{i}^{\prime }\gamma_{j}$ for counts and $\Phi^{-1}\left(\mu_{ij}\right)=\alpha_{i}+\beta_{0j}+u_{i}^{\prime }\gamma_{j}$ for presence-absence data, where *α*_*i*_ is a random effects assumed to follow a normal distribution with zero mean and variance 0.25. We fitted these models with random row effects using TMB only. The reason for this is that the plain R implementations of [21] do not cater for random row effects, and other simulations had already demonstrated that the TMB implementation is more computationally efficient.

Note that the first simulation setup, based on a dataset with a large sample size, varied *n*, while the second simulation setup, based on a dataset with a species rich community (large *m*), varied *m*. Hence we looked at the effects of varying each of sample size and of number of responses, but do so one simulation at a time. These simulations were computationally intensive, with a total running time across all simulations of 5 weeks on a Intel Xeon E7-8837 (2.67GHz) processor with 25 CPUs.

### 4.2 Overdispersed counts

**Fig 1:**
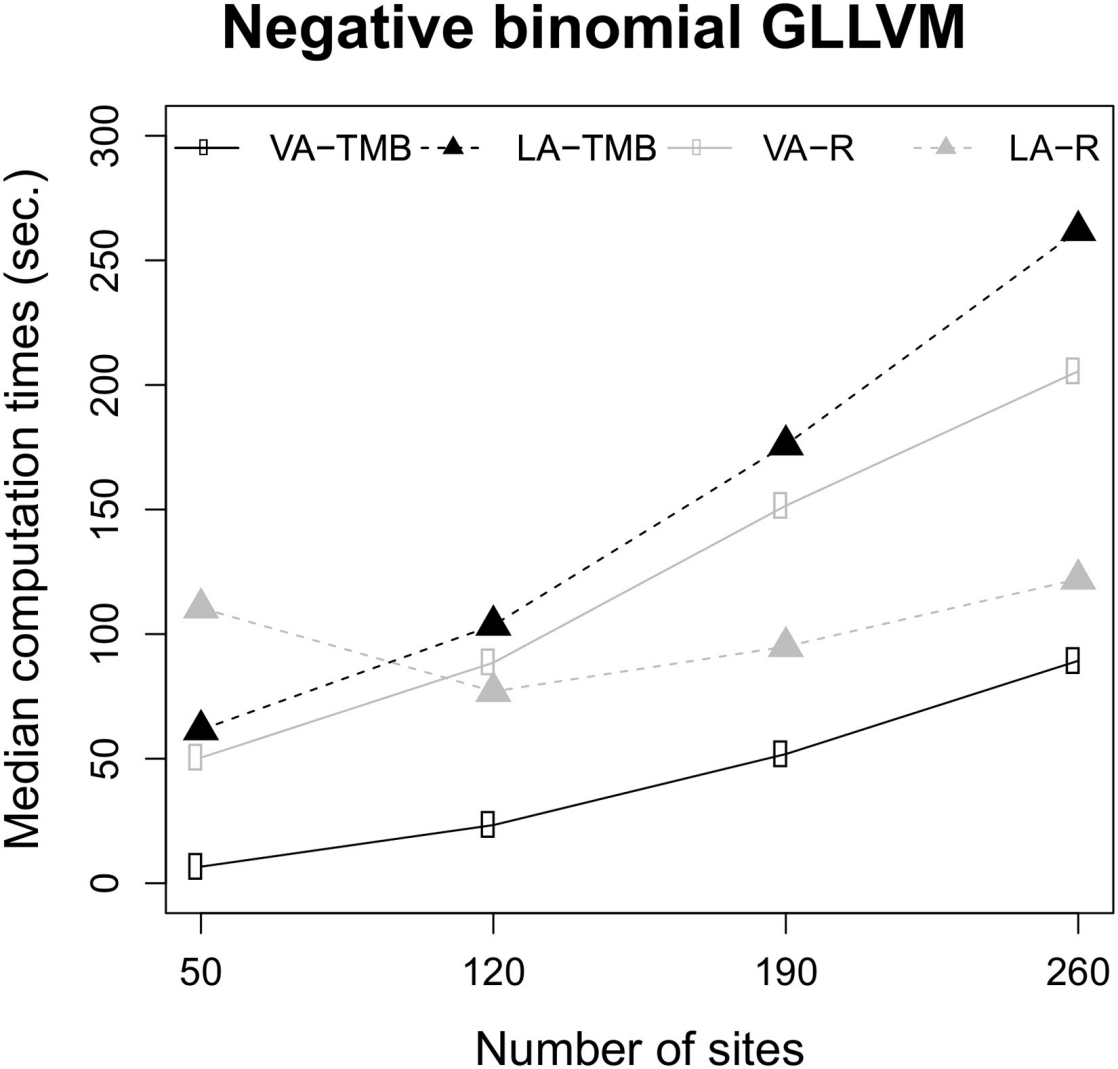
Median computation times for negative binomial GLLVMs. Times for the plain R (gray) and the TMB implementations (black) for the variational approximation (VA, solid line) method and the Laplace approximation (LA, dashed line) method for a negative binomial GLLVM with two covariates and two latent variables. The simulation setup was based on testate amoebae data.

We being by presenting the results from negative binomial GLLVM under the first simulation design, and compared variational approximation and Laplace approximation methods implemented with and without TMB, using the starting value method res; see Section 4.4 for the reason behind this choice of starting value approach. Fig 1 plots the median computation times, and demonstrates that the variational approximation method implemented using TMB was substantially faster than the other estimation methods. The TMB implementation of the Laplace approximation method was also faster than the plain R implementation for the smallest sample size.

The results in Table 1 suggest that the advantages in computation time did not come at the cost of estimation and inferential accuracy. In fact, the average biases across all species and root mean squared errors tended to be smaller for the variational approximation method compared to the Laplace approximation method. With very small *n*, the differences between the two approximation methods were particularly noticeable. For both methods, the estimates for log-dispersion parameters were comparably biased when the sample size was very small. When the sample size increased, the variational approximation method in particular performed better, with differences between the two variational approximation implementations becoming very small. For the Laplace approximation method, although the differences in average biases were small, the differences in coverage probabilities and mean confidence interval widths were comparably larger than its variational counterpart. Furthermore, the implementation which did not use TMB tended to provide overly narrow confidence intervals for almost all parameters.

**Table 1:** Average biases, root mean squared errors (RMSE), coverage probabilities of 95% confidence intervals and mean confidence interval widths (CI) for negative binomial GLLVM estimates based on the plain R and the TMB implementations for the variational approximation and the Laplace approximation methods. The true model parameters were obtained by fitting a negative binomial GLLVM with two environmental covariates for the testate amoebae data with counts of *m* = 48 species recorded at *n* = 50, 120, 190 and 260 sites. Parameter *β*_0_ refers to the species specific intercepts, *β*_*pH*_ and *β*_*temp*_ to the coefficients of water pH and water temperature and log *ϕ* to the log transformed dispersion parameters.

*n*		VA-TMB				LA-TMB			
		Bias	RMSE	Cover	CI	Bias	RMSE	Cover	CI
50	*β*_0_	-0.32	0.85	0.94	3.09	-0.92	2.24	0.93	5.14
	*β*_*pH*_	-0.03	0.63	0.95	2.44	0.01	0.90	0.95	2.94
	*β*_*temp*_	0.02	0.73	0.93	2.76	-0.05	0.97	0.93	3.31
	log *ϕ*	-0.38	0.67	0.92	2.35	-2.80	5.12	0.95	76.72
120	*β*_0_	-0.05	0.49	0.94	1.78	-0.33	0.99	0.95	2.53
	*β*_*pH*_	-0.04	0.40	0.95	1.55	-0.01	0.46	0.95	1.67
	*β*_*temp*_	0.02	0.37	0.96	1.48	0.00	0.46	0.96	1.65
	log *ϕ*	-0.06	0.36	0.94	1.48	-0.59	1.57	0.95	5.13
190	*β*_0_	0.03	0.40	0.92	1.36	-0.19	0.62	0.96	1.80
	*β*_*pH*_	-0.04	0.32	0.95	1.20	-0.01	0.34	0.95	1.27
	*β*_*temp*_	0.01	0.30	0.97	1.24	0.00	0.36	0.96	1.34
	log *ϕ*	0.02	0.30	0.93	1.16	-0.24	0.62	0.95	1.81
260	*β*_0_	0.07	0.36	0.91	1.15	-0.13	0.46	0.96	1.46
	*β*_*pH*_	-0.04	0.27	0.96	1.05	-0.02	0.29	0.96	1.10
	*β*_*temp*_	0.01	0.25	0.97	1.05	0.01	0.29	0.97	1.11
	log *ϕ*	0.06	0.28	0.91	0.99	-0.15	0.36	0.95	1.24
		VA-R				LA-R			
50	*β*_0_	-0.31	0.85	0.95	3.15	-0.94	2.34	0.84	4.60
	*β*_*pH*_	-0.03	0.63	0.95	2.48	-0.00	0.86	0.72	2.18
	*β*_*temp*_	0.02	0.73	0.94	2.80	-0.05	0.98	0.67	2.19
	log *ϕ*	-0.38	0.67	0.93	2.42	-1.44	2.39	0.51	3.27
120	*β*_0_	-0.05	0.49	0.95	1.79	-0.31	0.97	0.89	2.17
	*β*_*pH*_	-0.04	0.40	0.95	1.56	-0.02	0.48	0.79	1.54
	*β*_*temp*_	0.02	0.37	0.96	1.49	0.00	0.46	0.81	1.61
	log *ϕ*	-0.06	0.36	0.95	1.49	-0.40	0.86	0.56	0.85
190	*β*_0_	0.03	0.40	0.92	1.37	-0.18	0.60	0.91	1.55
	*β*_*pH*_	-0.04	0.32	0.95	1.20	-0.02	0.39	0.77	1.22
	*β*_*temp*_	0.01	0.30	0.97	1.24	-0.00	0.39	0.79	1.30
	log *ϕ*	0.02	0.30	0.93	1.17	-0.21	0.48	0.58	0.63
260	*β*_0_	0.07	0.36	0.91	1.15	-0.12	0.45	0.89	1.26
	*β*_*pH*_	-0.04	0.27	0.96	1.05	-0.03	0.39	0.71	1.04
	*β*_*temp*_	0.01	0.25	0.97	1.05	0.01	0.34	0.77	1.11
	log *ϕ*	0.06	0.28	0.91	0.99	-0.13	0.35	0.59	0.53

In order to evaluate the performance of the estimated latent variable loadings, ${\hat{\gamma }}_{j}$, and predicted latent variables, ${\hat{u}}_{i}$, we list in Table 2 the mean Procrustes errors between the estimated and the true values ([28], Chapter 8.4). These are scaled according to the sample size and number of species to make comparisons easier. Results indicated that for small *n*, compared to the Laplace approximation method, the variational approximation method produced smaller Procrustes errors for both latent variables and loadings. As expected, the difference between Procrustes errors based on different methods decreased when *n* increased.

**Table 2:** Scaled mean Procrustes errors of predicted latent variables and estimated latent variable loadings for negative binomial GLLVM estimates based on the plain R and the TMB implementations for the variational approximation and the Laplace approximation methods. The true model parameters were obtained by fitting a negative binomial GLLVM for the testate amoebae data with counts of *m* = 48 species recorded at *n* = 50, 120, 190 and 260 sites.

*n*	VA-TMB		LA-TMB		VA-R		LA-R	
	LVs	Loadings	LVs	Loadings	LVs	Loadings	LVs	Loadings
50	0.256	0.346	0.296	0.497	0.256	0.347	0.328	0.489
120	0.198	0.198	0.208	0.296	0.198	0.198	0.219	0.276
190	0.185	0.147	0.189	0.213	0.185	0.148	0.213	0.195
260	0.177	0.118	0.179	0.150	0.177	0.119	0.216	0.135

In addition to the results presented in Tables 1 and 2, we also evaluated the acurracy of competing models by adapting the variation explained based on cross-validation (denoted here as VE), as proposed in [38, 39], for our text with simulated binary and count data. Specifically, for each simulation setup we compared the predictive performance of the correponding GLLVM to the null model i.e, a model including only an species-specific intercept only, using the formula

$$\begin{array}{c} VE_{k}=1-\frac{\sum_{i=1}^{n}\sum_{j=1}^{m}\left|{\hat{\mu }}_{ij}^{\left(k\right)}-\mu_{ij}\right|}{\sum_{i=1}^{n}\sum_{j=1}^{m}\left|{\hat{\mu }}_{ij,null}^{\left(k\right)}-\mu_{ij}\right|}, \end{array}$$

where for the *k*th simulated dataset with *k* = 1, …, 500, the quantities ${\hat{\mu }}_{ij}^{\left(k\right)}$ and ${\hat{\mu }}_{ij,null}^{\left(k\right)}=g^{-1}({\hat{\beta }}_{0j})$ denote the predicted means from the fitted GLLVM and from a null model, respectively. The true means, which were used to generate the training datasets, are denoted by *μ*_*ij*_. Because we are using simulated data and therefore can generate multiple training datasets, as opposed to a real application where we only have the one realized dataset, then there is less motivation to use cross-validation when calculating VE i.e, the natural variation across folds can be well accounted by the natural variation across simulated datasets. Also, note because we are working with discrete data, then we choose to calculate VE based on the predicted mean scale *μ*_*ij*_ rather than on the response scale. The median VE values for negative binomial GLLVMs fitted to counts simulated based on amoebae dataset are listed in Table 3. The results indicate that the predictive accuracy improves as the number of sites increases. The accuracy is slightly higher when the variational approximation method is used. Further, when *n* > 50, the Laplace approximation method using the R implementation gives clearly lower VE values than the method using the TMB implementation.

**Table 3:** Median VE values of negative binomial GLLVMs for 500 simulated datasets using the plain R and the TMB implementations for the variational approximation and the Laplace approximation methods. The datasets were based on a negative binomial GLLVM fitted for the testate amoebae data with counts of *m* = 48 species recorded at *n* = 50, 120, 190 and 260 sites.

*n*	VA-TMB	LA-TMB	VA-R	LA-R
50	0.27	0.19	0.27	0.21
120	0.48	0.43	0.42	0.29
190	0.53	0.50	0.53	0.35
260	0.56	0.54	0.56	0.39

The simulation results based on the negative binomial GLLVMs fitted for Indonesian bird data, with and without random row effect are given in S2 Appendix. Broadly speaking, they returned similar conclusions to those reported above. However, for both methods the log standard deviations of the random row effects were highly biased when the number of species was *m* = 30 but accuracy improved substantially with larger *m*. In addition, the predictive accuracy improves when the number of species increases.

### 4.3 Binary responses

Below we use the second simulation design to compare the performance of both approximation methods implemented with and without TMB for GLLVMs with binary responses. As previously, starting values obtained via the res method.

**Fig 2:**
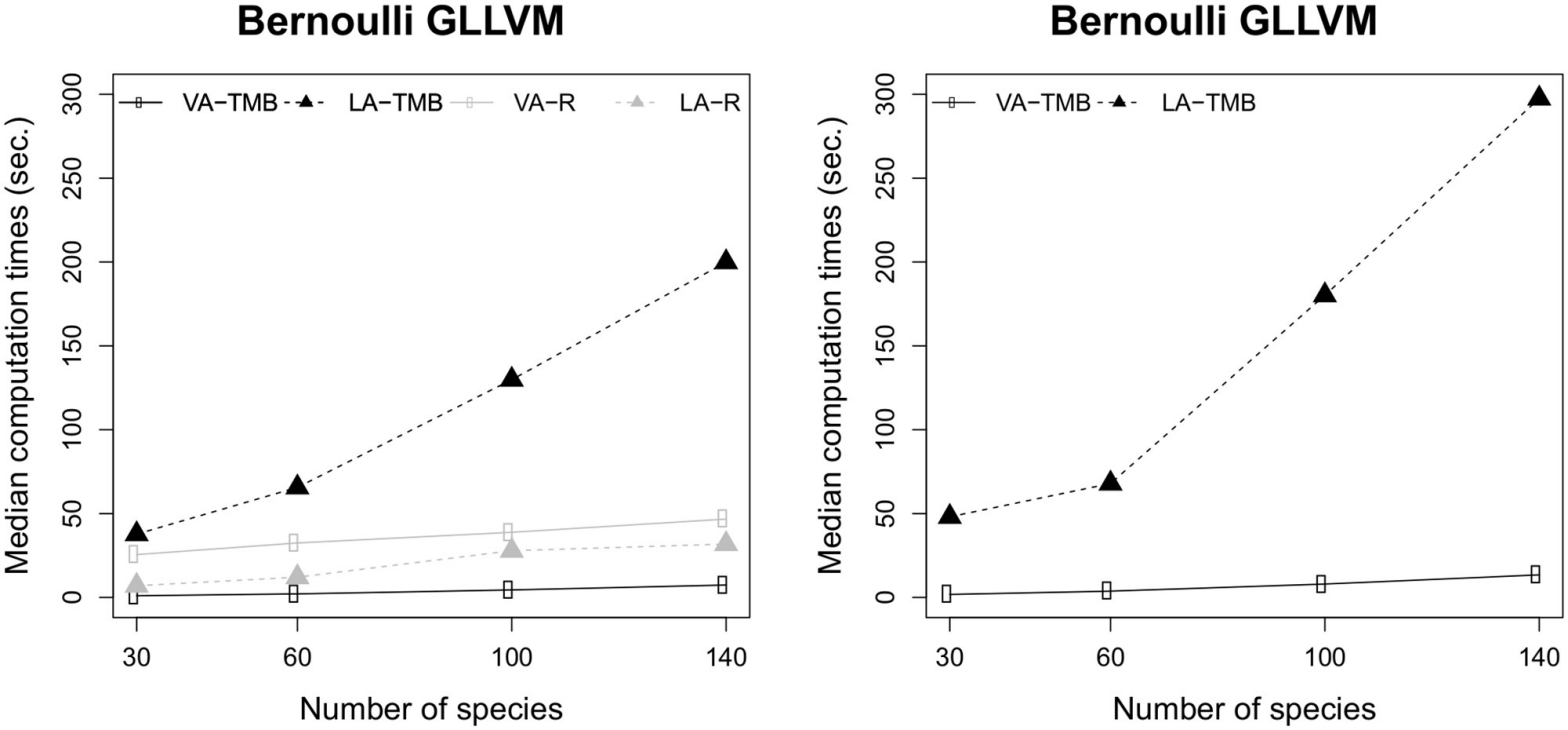
Median computation times for Bernoulli GLLVMs. Times for the plain R (gray) and the TMB implementations (black) for the variational approximation (VA, solid line) method and the Laplace approximation (LA, dashed line) method for a Bernoulli GLLVM with two latent variables. The left plot is for the model without row effects and right one with random row effects. The simulation setup was based on the Indonesian birds data.

Fig 2 presents the computation times of various methods used to fit GLLVMs to binary responses. Similar to the simulation involving overdispersed counts, the variational approximation method implemented using TMB was substantially faster than all the other methods for all considered cases. It was also interesting to note that the median computation times for the Laplace approximation method implemented using TMB scaled very poorly with increasing *n*.

Table 4 lists the average biases, root mean squared errors, 95% coverage probabilities and mean confidence interval widths for estimates of the GLLVM without random row effects from different estimation methods. As in the case of overdispersed counts, the number of species did not have much effect on the estimates of species specific intercepts, *β*_0_. The variational approximation method performed better overall in each of the considered cases, producing less biased estimates, smaller root mean squared errors and coverage probabilities closer to the nominal coverage level of 95%. By contrast, the estimates based on the Laplace approximation were severely biased, especially when the sample size was small. When *m* increased, the biases became smaller for both methods and the coverage probabilities approached to the nominal 95% level when the Laplace approximation were used. Results for the scaled mean Procrustes errors in Table 5 showed that errors were tended to be smaller when the variational approximation method was used in estimation compared to the Laplace approximation method. As in the simulation settings with overdispersed counts, the mean Procrustes errors for latent variables predictions decreased with an increasing number of species *m*.

**Table 4:** Average biases, root mean squared errors (RMSEs), coverage probabilities of 95% confidence intervals and mean confidence intervals widths (CI) for GLLVM estimates based on the plain R and the TMB implementations for the variational approximation and the Laplace approximation methods. The true model parameters were obtained by fitting a Bernoulli GLLVM with probit link function for the Indonesian birds data with presence-absences of *m* = 30, 60, 100 and 140 species recorded at *n* = 37 sites.

*m*		VA-TMB				LA-TMB			
		Bias	RMSE	Cover	CI	Bias	RMSE	Cover	CI
30	*β*_0_	0.05	0.29	0.93	1.27	-4.43	18.24	0.73	5.22
60	*β*_0_	-0.03	0.30	0.98	1.55	-0.22	7.77	0.89	5.23
100	*β*_0_	-0.03	0.35	0.96	1.55	-0.05	5.37	0.92	3.19
140	*β*_0_	-0.03	0.39	0.96	1.57	-0.04	1.04	0.92	2.07
		VA-R				LA-R			
30	*β*_0_	0.05	0.29	0.93	1.27	-0.01	0.46	0.81	1.31
60	*β*_0_	-0.03	0.30	0.98	1.54	-0.14	0.67	0.83	1.57
100	*β*_0_	-0.03	0.35	0.96	1.55	-0.12	0.95	0.84	1.69
140	*β*_0_	-0.03	0.39	0.96	1.56	-0.10	0.94	0.83	1.49

**Table 5:** Scaled mean Procrustes errors of predicted latent variables and estimated latent variable loadings for GLLVM estimates based on the plain R and the TMB implementations for the variational approximation and the Laplace approximation methods. Values are scaled with the number of sites and number of species for comparisons. The true model parameters were obtained by fitting a Bernoulli GLLVM with probit link function for the Indonesian birds data with presence-absences of *m* = 30, 60, 100 and 140 species recorded at *n* = 37 sites.

*m*	VA-TMB		LA-TMB		VA-R		LA-R	
	LVs	Loadings	LVs	Loadings	LVs	Loadings	LVs	Loadings
30	0.556	0.122	0.615	0.140	0.556	0.122	0.615	0.173
60	0.185	0.098	0.204	0.160	0.185	0.098	0.204	0.141
100	0.129	0.095	0.144	0.130	0.129	0.095	0.144	0.139
140	0.098	0.091	0.109	0.121	0.098	0.091	0.109	0.126

Variation explained was computed for Bernoulli GLLVMs as in Section 4.2, and the median VE values are listed in Table 6. Based on the results, differences in predictive accuracies improve with increasing *m*. The variance explained is substantially lower for the Laplace approximation method compared to the variational approximation method when number of species is small, but equally good for large *m*.

**Table 6:** Median VE values of Bernoulli GLLVMs for 500 simulated datasets using the plain R and the TMB implementations for the variational approximation and the Laplace approximation methods. The datasets were based on a Bernoulli GLLVM with probit link function fitted for the Indonesian birds data with presence-absences of *m* = 30, 60, 100 and 140 species recorded at *n* = 37 sites.

*m*	VA-TMB	LA-TMB	VA-R	LA-R
30	0.23	0.08	0.24	0.08
60	0.30	0.28	0.30	0.26
100	0.34	0.30	0.31	0.31
140	0.36	0.35	0.36	0.36

Supporting information S2 Appendix reports results for simulations based on the Indonesian bird dataset with a random row effect, and for simulations based on the testate amoebae data when converted to presence-absence data. Results were broadly similar to those reported for *β*_0_ in Table 4, with the variational approximation leading to more accurate and precise estimates, while the Laplace approximation method tended to produce severely biased estimates particularly at small sample sizes. For both approximation methods, the log standard deviations of the random row effects were biased when the number of species *m* was small.

### 4.4 Starting value comparisons

To study the sensitivity of model fitting results to starting values, we compared the performances of four starting value selection strategies explained in section 3.2. As a global performance measure, we used the log-likelihood values obtained from res3 as a reference level, and compared differences between this and the three other methods (res, zero, random).

**Fig 3:**
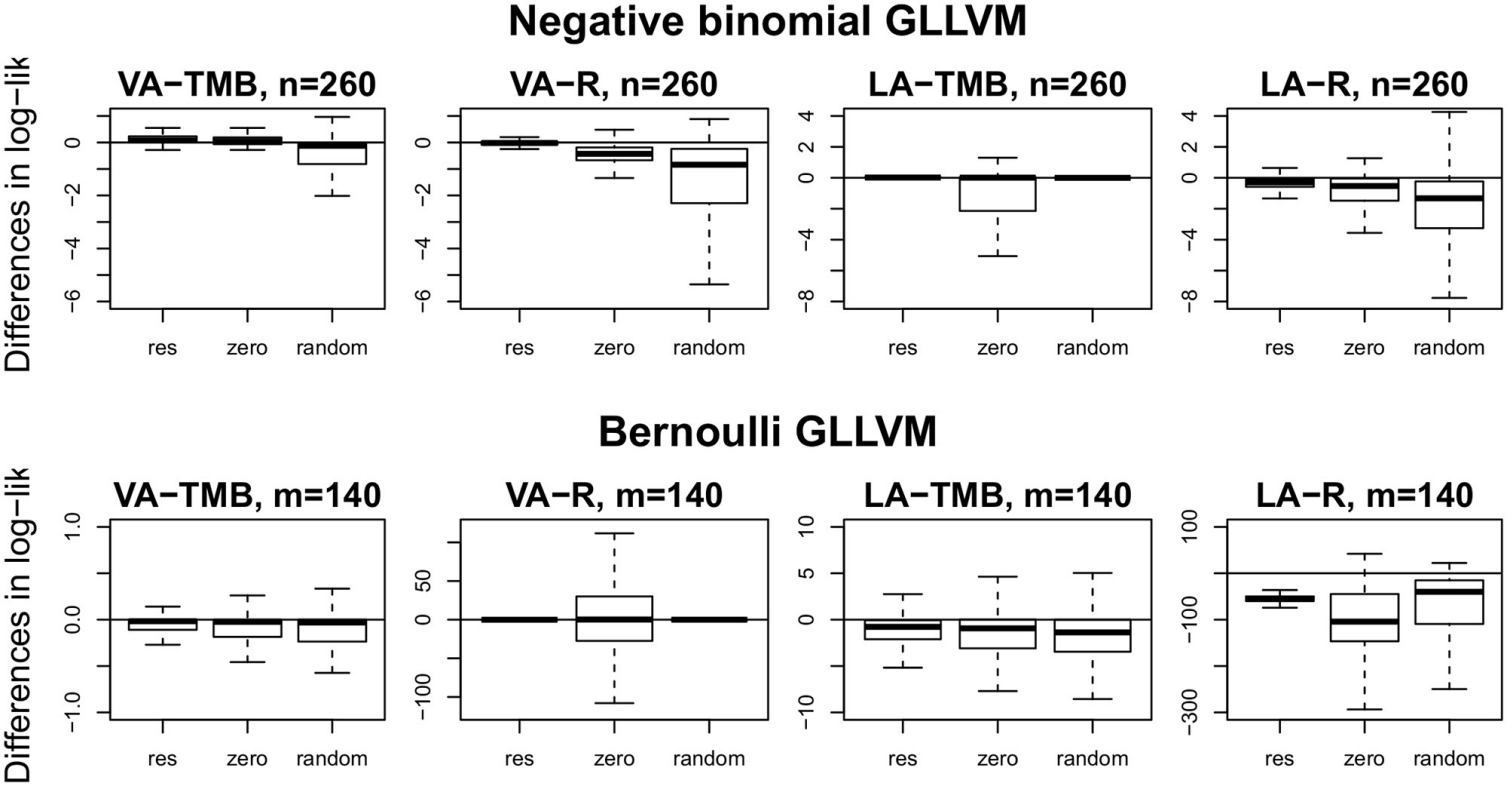
Differences in log-likelihood value when strategies res, zero and random are compared to res3. The true models were based on negative binomial GLLVM fitted for the Testate amoebae data with *n* = 260 sites and Bernoulli GLLVM fitted for the Indonesian bird data with *m* = 140 species. A negative value means that performance of the corresponding starting value strategy is worse than that of res3. Notice that columns have different scales.

Boxplots of the differences in log-likelihood values are given in Fig 3 for negative binomial GLLVMs fitted for the Testate amoebae data with *n* = 260 sites and *m* = 48 species, and for Bernoulli GLLVMs fitted for the Indonesian bird data with *n* = 37 sites and *m* = 140 species. When the TMB implementation of the variational approximation method was used the differences between the log-likelihood values based on res3 and the other three methods were relatively small. The biggest differences were seen when the Laplace approximation method and the variational approximation method were implemented without TMB and applied to binary data. The full results with simulated datasets of different sizes may be found in S3 Appendix. In all of the considered cases, res3 and res were consistently among the best starting values strategies giving the highest log-likelihood values, while the performances of zero and random depended strongly on the simulation setup.

In addition to the differences in log-likelihood values illustrated in Fig 3 for Bernoulli GLLVMs and in S3 Appendix for negative binomial GLLVMs, we also list for binary responses of the Indonesian bird data the average biases, root mean squared errors, 95% coverage probabilities and mean confidence interval widths for species specific intercept estimates as well as scaled mean Procrustes errors of predicted latent variables and estimated latent variable loadings for all methods included in comparisons in S3 Appendix.

Overall, these findings suggest that res and res3 were the best strategies for choosing starting values. All methods res, zero and random have been implemented as different options (with the same names) in the R package gllvm with res as the default.

## 5 Discussion

In this article, we studied two closed form approximations (the Laplace approximation and variational approximation) for the marginal log-likelihood of a generalized linear latent variable model. We showed how the closed form approximations can be implemented efficiently using automatic optimization techniques implemented in R with the help of the package TMB. In addition, a new method for choosing the starting values for our estimation algorithms was proposed. The performances of the two approximation methods and different starting values strategies were compared using several simulation studies for overdispersed count and binary data, which are often encountered in biological and ecological studies. Results indicated that for both response types the variational approximation implementations tended to outperform the Laplace approximation implementations, both in terms of computation speed and estimation and inferential accuracy. These findings are congruent with the results of Hui *et al*. [7], where the performance of the variational approximation method was compared to the Laplace approximation method and the MCEM algorithm for count and binary data, and also to Gauss-Hermite Quadrature in the case of binary data. However, more comprehensive comparisons between the variational approximation method and other estimation methods, eg. the Gauss-Hermite Quadrature, would be useful and interesting in the future.

The Laplace approximation method implemented without automatic optimization showed the poorest performance in all of the considered cases. The differences between the TMB and R implementations, especially with the Laplace approximation, are most likely due to the differences in the optimization algorithms. In the R implementation we used a block-coordinate optimization in which we cycled between iterative updates of one of regression coefficients, latent variables and nuisance parameters, until convergence. We postulate that this led to a less targeted exploration of the parameter space with an increased chance of getting trapped in a local maximum. In the case of binary data, the variational approximation implementations performed substantially better than their Laplace approximation counterparts. This supports earlier findings that the Laplace approximation method often performs poorly with highly discrete responses [40].

All simulation studies further showed that we can obtain more accurate predictions of the latent variables by increasing the number of species, *m*. For the Laplace method this is explained by the asymptotic error, which is known to be of order *O*(*m*^−1^) [41]. Although not proven here, we conjecture that for the variational approximation method, the asymptotic error is *O*(*m*^−1^); see also the heuristic proof of consistency in [19]. However, more accurate estimates for model parameters can be obtained only by increasing the sample size, *n*.

Another way to of obtaining more accurate estimates and inferential for the parameters in a GLLVM is by introducing structure that allows us to borrow strength across species (response) in order to estimate regression and/or loading parameters. Not only does this decrease the number of parameters in the model, it also means that these new parameters are a function of *n* and *m*, and thus accuracy of their estimation and inference should improve when either the number of sites and/or species increases. An examples is using functional traits in order to mediate the species environment relationships (sometimes called a “fourth corner model”, [42]): the resulting fourth corner coefficients parameters are then common to all species and estimation should improve as both a function of *n* and *m* both. Fourth corner models with latent variables can also be fitted using the R package gllvm, which implements both the Laplace and variational approximation methods.

Comparison of computation times clearly indicate that the TMB implementation of the variational approximation method is much faster than that both implementations of the Laplace approximation, with the difference becoming greater when the data are higher-dimensional. There are a number of reasons for this: first, we specified the variational approximation of the likelihood directly in C++, while for a Laplace approximation we only specified the integrand, and asked the TMB package to use automatic differentiation to calculate a Laplace approximation. This automation of the Laplace approximation offers considerable flexibility, and makes it relatively easy to fit some quite complex models, because the joint likelihood in the integrand is usually relatively easy to derive. However, it seems that not specifying a fully closed form (approximated) marginal log-likelihood comes at a computational cost. Another reason for a difference in computational time is that all variational parameters are handled like fixed parameters, which makes estimation faster than dealing with random effects. The other possible reason for more rapid growth in computation time for the Laplace approximation method, when *m* increases, comes from the complexity of the approximation itself, where there is a term $\log\det\{G(\Psi ,{\hat{u}}_{i}^{*})\}$, where $G(\Psi ,{\hat{u}}_{i}^{*})$ has dimension *m*, and so computing its determinant has a complexity that grows at a rate *O*(*m*^3^).

Overall, our findings suggest present a strong case for the use of the variational approximation method as a primary method for performing likelihood based estimation and inference in GLLVMs. Because it is relatively accurate and very quick, variational approximation on TMB provides a platform for upscaling analyses to large datasets. To date we have used the software to fit a dataset of size 174 × 985 in 61 minutes. In future work, we plan to generalize GLLVMs, as well as the gllvm package, so that it can handle spatial and or temporal correlation inherent in the data, as well as offer some data-driven forms of order and variable selection (see for example [43]).

## Supporting information

S1 Appendix Proof of the variational approximation of the likelihood of GLLVMs. (PDF)

S2 Appendix Additional simulation results. Results of the negative binomial GLLVM simulation for the Indonesian birds data and the Bernoulli GLLVM simulation for the testate amoebae data.
(PDF)

S3 Appendix Full results for the starting value comparisons. (PDF)

S1 File R code for simulations. (R)

S2 File Amoebae data. (ZIP)
