# Supplementary material for: Efficient estimation of generalized linear latent variable models
Source: PLoS One. 2019 May 1;14(5):e0216129. doi: 10.1371/journal.pone.0216129 (PMC6493759; doi:10.1371/journal.pone.0216129)
Supplement: S1 Appendix — (PDF) [file pone.0216129.s001.pdf]

S1 Appendix. Proof of the variational approximation of the likelihood of GLLVMs

Assume that the responses come from the exponential family of distributions with density $f(y_{ij}|\boldsymbol{u}_i^*, \boldsymbol{\Psi}) = \exp\{(y_{ij}\eta_{ij} - b(\eta_{ij}))/\phi_j + c(y_{ij}, \phi_j)\}$. The variational approximation for the marginal log-likelihood is then obtained as follows

$$\begin{aligned}
\underline{\ell}(\boldsymbol{\Psi}, \boldsymbol{\xi}) &= \sum_{i=1}^{n} \int \log\left\{\frac{f(\boldsymbol{y}_i|\boldsymbol{u}_i^*, \boldsymbol{\Psi}) f(\boldsymbol{u}_i^*)}{q^*(\boldsymbol{u}_i^*|\boldsymbol{\xi})}\right\} q^*(\boldsymbol{u}_i^*|\boldsymbol{\xi}) d\boldsymbol{u}_i^*, \\
&= \sum_{i=1}^{n} \int \{\log f(\boldsymbol{y}_i|\boldsymbol{u}_i^*, \boldsymbol{\Psi}) + \log f(\boldsymbol{u}_i^*) - \log q^*(\boldsymbol{u}_i^*|\boldsymbol{\xi})\} q^*(\boldsymbol{u}_i^*|\boldsymbol{\xi}) d\boldsymbol{u}_i^*, \\
&= \sum_{i=1}^{n} \Big(E_{q^*}\{\log f(\boldsymbol{y}_i|\boldsymbol{u}_i^*, \boldsymbol{\Psi})\} + E_{q^*}\{\log f(\boldsymbol{u}_i^*)\} + E_{q^*}\{-\log q^*(\boldsymbol{u}_i^*|\boldsymbol{\xi})\}\Big),
\end{aligned}$$

where E_{q*} is expectation with respect to variational density q^*. Expectation $E_{q^*}\{-\log q^*(\boldsymbol{u}_i^*|\boldsymbol{\xi})\}$ is the definition to the entropy of $q^*(\boldsymbol{u}_i^*|\boldsymbol{\xi})$ which equals to $\log\det(2\pi e\boldsymbol{A}_i)/2$. When we omit all quantities constant with respect to the parameters, the above equals to

$$\begin{aligned}
\underline{\ell}(\boldsymbol{\Psi}, \boldsymbol{\xi}) =& \sum_{i=1}^{n}\sum_{j=1}^{m}\left\{\frac{y_{ij}\tilde{\eta}_{ij} - E_{q^*}\{b(\eta_{ij})\}}{\phi_j} + c(y_{ij}, \phi_j)\right\} \\
&+ \frac{1}{2}\sum_{j=1}^{n}\left\{\log\det\boldsymbol{A}_i - E_{q^*}\left\{\boldsymbol{u}_i^{*\prime}\boldsymbol{C}_{\sigma^2}^{-1}\boldsymbol{u}_i^* + \log\det(\boldsymbol{C}_{\sigma^2})\right\}\right\} \\
=& \sum_{i=1}^{n}\sum_{j=1}^{m}\left\{\frac{y_{ij}\tilde{\eta}_{ij} - E_{q^*}\{b(\eta_{ij})\}}{\phi_j} + c(y_{ij}, \phi_j)\right\} \\
&+ \frac{1}{2}\sum_{i=1}^{n}\Big(\log\det(\boldsymbol{A}_i) - \mathrm{tr}(\boldsymbol{C}_{\sigma^2}^{-\frac{1}{2}}\boldsymbol{A}_i\boldsymbol{C}_{\sigma^2}^{-\frac{1}{2}}) - \boldsymbol{a}_i'\boldsymbol{C}_{\sigma^2}^{-1}\boldsymbol{a}_i - \log\det(\boldsymbol{C}_{\sigma^2})\Big) \\
=& \sum_{i=1}^{n}\sum_{j=1}^{m}\left\{\frac{y_{ij}\tilde{\eta}_{ij} - E_{q^*}\{b(\eta_{ij})\}}{\phi_j} + c(y_{ij}, \phi_j)\right\} \\
&+ \frac{1}{2}\sum_{i=1}^{n}\left(\log\det(\boldsymbol{A}_i) - \mathrm{tr}(\boldsymbol{C}_{\sigma^2}^{-1}\boldsymbol{A}_i) - \boldsymbol{a}_i'\boldsymbol{C}_{\sigma^2}^{-1}\boldsymbol{a}_i - \log\det(\boldsymbol{C}_{\sigma^2})\right),
\end{aligned}$$

where $\tilde{\eta}_{ij} = a_{\alpha i} + \beta_{0j} + \boldsymbol{x}_i'\boldsymbol{\beta}_j + \boldsymbol{a_u}_i'\gamma_j$, $\boldsymbol{C}_{\sigma^2}$ is block diagonal matrix of σ^2 and $\boldsymbol{I}_d$ and $\boldsymbol{u}_i^* = (\alpha_i, \boldsymbol{u}_i')'$. Notation $\boldsymbol{C}_{\sigma^2}^{-1/2}$ is the square root of $\boldsymbol{C}_{\sigma^2}^{-1}$ which means $\boldsymbol{C}_{\sigma^2}^{-\frac{1}{2}}\boldsymbol{C}_{\sigma^2}^{-\frac{1}{2}}{=}\boldsymbol{C}_{\sigma^2}^{-1}$. This operation is possible when $\boldsymbol{C}_{\sigma^2}$ is diagonal matrix.
