# Supplementary material for: Efficient estimation of generalized linear latent variable models
Source: PLoS One. 2019 May 1;14(5):e0216129. doi: 10.1371/journal.pone.0216129 (PMC6493759; doi:10.1371/journal.pone.0216129)
Supplement: S2 Appendix — Results of the negative binomial GLLVM simulation for the Indonesian birds data and the Bernoulli GLLVM simulation for the testate amoebae data. (PDF) [file pone.0216129.s002.pdf]

S2 Appendix. Additional simulation results

The Indonesian birds data: Negative binomial GLLVM

Table S2.1. Average biases, root mean squared errors (RMSEs), coverage probabilities of 95% confidence intervals and mean confidence interval widths (CI) for GLLVM estimates based on the plain `R` and the `TMB` implementations for the variational approximation and the Laplace approximation methods. The true models were based on negative binomial GLLVMs fitted for Indonesian bird data with $m = 30, 60, 100$ and 140 species recorded at $n = 37$ sites.

		VA-`TMB`				LA-`TMB`			
m		Bias	RMSE	Cover	CI	Bias	RMSE	Cover	CI
30	β_0	0.18	0.61	0.88	1.93	-0.27	0.93	0.92	3.01
	$\log \phi$	0.03	0.72	0.91	2.88	-4.03	6.78	0.95	191.89
60	β_0	-0.02	0.52	0.95	2.10	-0.24	0.85	0.96	2.79
	$\log \phi$	-0.08	0.68	0.96	3.06	-2.69	4.83	0.97	98.18
100	β_0	-0.11	0.49	0.96	1.95	-0.20	0.71	0.96	2.29
	$\log \phi$	-0.19	0.67	0.96	2.99	-1.78	3.58	0.98	56.78
140	β_0	-0.15	0.51	0.96	1.97	-0.20	0.71	0.96	2.24
	$\log \phi$	-0.24	0.69	0.96	3.06	-1.44	2.85	0.98	25.86
		VA-R				LA-R			
30	β_0	0.18	0.61	0.88	1.93	-0.37	1.11	0.86	2.66
	$\log \phi$	0.03	0.72	0.93	3.03	-1.59	2.51	0.96	41.64
60	β_0	-0.02	0.52	0.95	2.10	-0.42	1.15	0.89	2.77
	$\log \phi$	-0.07	0.68	0.96	3.05	-1.09	1.80	0.97	26.85
100	β_0	-0.10	0.48	0.96	1.95	-0.33	0.90	0.89	2.23
	$\log \phi$	-0.18	0.66	0.96	2.98	-0.78	1.38	0.97	13.97
140	β_0	-0.14	0.51	0.96	1.97	-0.29	0.85	0.90	2.13
	$\log \phi$	-0.24	0.68	0.97	3.06	-0.74	1.33	0.97	12.53

Table S2.2. Median VE values of negative binomial GLLVMs for 500 simulated datasets using the plain `R` and the `TMB` implementations for the variational approximation and the Laplace approximation methods. The datasets were based on a negative binomial GLLVM fitted for the Indonesian birds data with counts of $m = 30, 60, 100$ and 140 species recorded at $n = 37$ sites.

m	VA-`TMB`	LA-`TMB`	VA-R	LA-R
30	0.01	-0.08	0.01	-0.07
60	0.25	0.21	0.25	0.21
100	0.35	0.32	0.35	0.28
140	0.37	0.35	0.36	0.31

Table S2.3. Mean Procrustes errors of predicted latent variables and estimated latent variable loadings for GLLVM estimates based on the plain `R` and the `TMB` implementations for the variational approximation and Laplace approximation methods. Values are scaled with the number of sites and number of species for comparisons. The true models were based on negative binomial GLLVMs fitted for the Indonesian birds data with counts of $m = 30, 60, 100$ and 140 species recorded at $n = 37$ sites.

	VA-`TMB`		LA-`TMB`		VA-R		LA-R	
m	LVs	Loadings	LVs	Loadings	LVs	Loadings	LVs	Loadings
30	0.475	0.121	0.513	0.149	0.473	0.121	0.517	0.149
60	0.171	0.174	0.183	0.226	0.172	0.174	0.187	0.222
100	0.128	0.203	0.133	0.255	0.128	0.202	0.138	0.258
140	0.077	0.181	0.081	0.245	0.077	0.180	0.086	0.243

Table S2.4. Average biases, root mean squared errors (RMSEs), coverage probabilities of 95% confidence intervals and mean confidence interval widths (CI) for GLLVM estimates based on the plain `R` and the `TMB` implementations for the variational approximation and the Laplace approximation methods. The true models were based on negative binomial GLLVMs fitted for the Indonesian bird data with $m = 30, 60, 100$ and 140 species recorded at $n = 37$ sites. Random row effects are included in the model.

		VA-`TMB`				LA-`TMB`			
m		Bias	RMSE	Cover	CI	Bias	RMSE	Cover	CI
30	β_0	0.25	0.67	0.85	1.92	-0.22	0.79	0.92	2.78
	$\log\phi$	0.11	0.72	0.92	2.86	-4.83	7.14	0.94	39.04
	$\log\sigma$	-3.55	19.14	0.95	27.29	-1.46	10.59	0.88	26.20
60	β_0	-0.02	0.47	0.95	2.03	-0.42	0.85	0.89	2.52
	$\log\phi$	0.08	0.67	0.92	2.76	-3.32	5.11	0.97	21.12
	$\log\sigma$	-0.40	1.30	0.88	1.40	-0.28	0.83	0.91	2.34
100	β_0	-0.06	0.45	0.95	1.81	-0.17	0.52	0.96	2.01
	$\log\phi$	-0.08	0.63	0.95	2.71	-2.44	4.00	0.97	34.65
	$\log\sigma$	-0.10	0.04	0.87	0.53	-0.20	1.11	0.89	0.51
140	β_0	-0.09	0.45	0.96	1.83	-0.18	0.51	0.96	1.98
	$\log\phi$	-0.17	0.63	0.96	2.81	-2.14	3.52	0.97	29.74
	$\log\sigma$	-0.07	0.02	0.88	0.50	-0.07	0.02	0.89	0.49

Table S2.5. Median VE values of negative binomial GLLVMs for 500 simulated datasets. Models were fitted using the variational approximation and the Laplace approximation methods. The datasets were based on a negative binomial GLLVM with random row effect fitted for the Indonesian birds data with counts of $m = 30, 60, 100$ and 140 species recorded at $n = 37$ sites.

m	VA-`TMB`	LA-`TMB`
30	0.10	0.00
60	0.33	0.31
100	0.46	0.43
140	0.48	0.46

Table S2.6. Mean Procrustes errors of predicted latent variables and estimated latent variable loadings for GLLVM estimates based on the variational approximation and the Laplace approximation methods. Values are scaled with the number of sites and number of species for comparisons. The true models were based on negative binomial GLLVMs with random row effect fitted for the Indonesian birds data with counts of $m = 30, 60, 100$ and 140 species recorded at $n = 37$ sites.

	VA		LA	
m	LVs	Loadings	LVs	Loadings
30	0.699	0.600	0.596	0.509
60	0.187	0.267	0.197	0.336
100	0.157	0.229	0.162	0.283
140	0.118	0.221	0.111	0.289

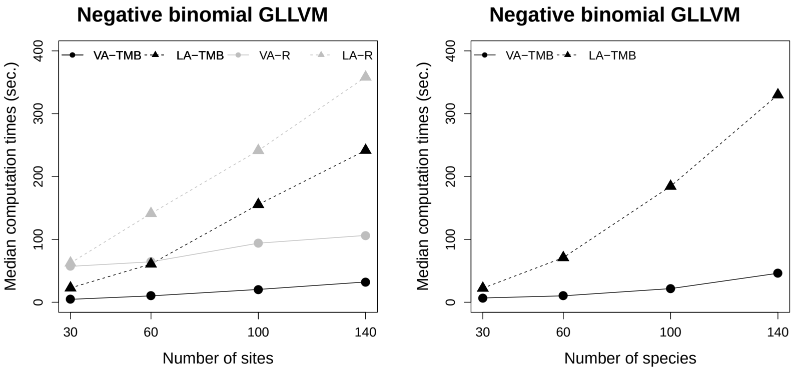

Fig S2.1. Median computation times for negative binomial GLLVMs. Median computation times for variational approximation (VA, solid line) and Laplace approximation (LA, dashed line) methods for a negative binomial GLLVM with two latent variables. The left plot is for the model without row effects and the right one with random row effects. The simulation setup is based on the Indonesian birds data.

The testate amoebae data: Bernoulli GLLVM

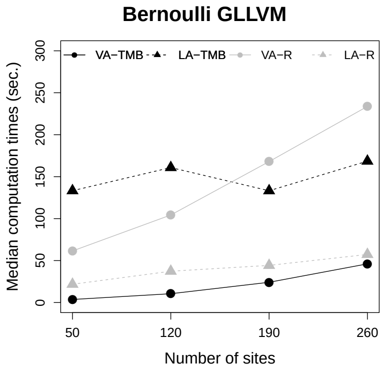

Fig S2.2. Median computation times for Bernoulli GLLVMs. Median computation times for the plain `R` (gray) and the `TMB` implementations (black) for the variational approximation (VA, solid line) method and the Laplace approximation (LA, dashed line) method for a Bernoulli GLLVM with two covariates and two latent variables. The simulation setup is based on the testate amoebae data.

Table S2.7. Average biases, root mean squared errors (RMSEs), coverage probabilities of 95% confidence intervals and mean confidence interval widths (CI) for GLLVM estimates based on variational approximation and Laplace approximation methods. The true models were based on Bernoulli GLLVMs with probit link function fitted for the testate amoebae data with presence-absences of $m = 48$ species recorded at $n = 50, 120, 190$ and 260 sites. Model includes two environmental covariates. Here parameter β_0 refers to the species specific intercepts, β_{pH} and β_{temp} to the coefficients of water pH and water temperature, respectively, and ϕ to the dispersion parameter.

		VA-TMB				LA-TMB			
n		Bias	RMSE	Cover	CI	Bias	RMSE	Cover	CI
50	β_0	-0.16	0.46	0.98	1.76	-9.38	26.62	0.79	9.99
	β_{pH}	-0.02	0.38	0.98	1.45	0.71	14.13	0.85	10.25
	β_{temp}	-0.01	0.40	0.97	1.59	0.43	10.11	0.87	10.52
120	β_0	-0.01	0.22	0.96	0.88	-0.14	0.33	0.92	1.74
	β_{pH}	-0.02	0.21	0.96	0.82	0.02	0.98	0.92	1.31
	β_{temp}	0.01	0.18	0.97	0.75	0.02	0.75	0.94	1.21
190	β_0	0.02	0.17	0.96	0.66	-0.08	0.20	0.96	0.92
	β_{pH}	-0.02	0.16	0.96	0.62	-0.01	0.19	0.95	0.71
	β_{temp}	0.00	0.15	0.97	0.62	0.01	0.18	0.96	0.71
260	β_0	0.03	0.15	0.94	0.55	-0.06	0.16	0.96	0.73
	β_{pH}	-0.02	0.13	0.96	0.54	0.00	0.15	0.96	0.61
	β_{temp}	-0.00	0.12	0.97	0.52	0.01	0.14	0.96	0.59
		VA-R				LA-R			
50	β_0	-0.16	0.46	0.98	1.73	-0.36	0.80	0.79	1.80
	β_{pH}	-0.02	0.37	0.98	1.44	-0.05	0.60	0.76	1.66
	β_{temp}	-0.02	0.39	0.97	1.57	0.03	0.54	0.75	1.61
120	β_0	-0.01	0.22	0.96	0.88	-0.12	0.34	0.73	0.78
	β_{pH}	-0.02	0.20	0.96	0.82	-0.02	0.29	0.78	0.82
	β_{temp}	0.01	0.18	0.97	0.75	0.03	0.26	0.79	0.80
190	β_0	0.02	0.17	0.96	0.66	-0.05	0.24	0.70	0.53
	β_{pH}	-0.02	0.16	0.96	0.62	-0.01	0.22	0.80	0.63
	β_{temp}	0.00	0.15	0.97	0.62	0.01	0.20	0.79	0.61
260	β_0	0.03	0.15	0.94	0.55	-0.03	0.20	0.68	0.43
	β_{pH}	-0.02	0.13	0.96	0.54	-0.00	0.19	0.79	0.52
	β_{temp}	-0.00	0.12	0.97	0.52	0.00	0.18	0.78	0.51

Table S2.8. Median VE values of Bernoulli GLLVMs for 500 simulated datasets using the plain `R` and the `TMB` implementations for the variational approximation and the Laplace approximation methods. The datasets were based on a Bernoulli GLLVM with probit link function fitted for the testate amoebae data with presence-absences of $m = 48$ species recorded at $n = 50, 120, 190$ and 260 sites.

n	VA-TMB	LA-TMB	VA-R	LA-R
50	0.34	0.16	0.27	0.14
120	0.52	0.45	0.52	0.41
190	0.58	0.54	0.58	0.48
260	0.60	0.57	0.60	0.46

Table S2.9. Mean Procrustes errors of predicted latent variables and estimated latent variable loadings for GLLVM estimates based on the plain `R` and the `TMB` implementations for the variational approximation and the Laplace approximation methods. Values are scaled with the number of sites and number of species for comparisons. The true models were based on Bernoulli GLLVMs with probit link function fitted for the testate amoebae data with presence-absences of $m = 48$ species recorded at $n = 50, 120, 190$ and 260 sites. Model includes two environmental covariates.

	VA-`TMB`		LA-`TMB`		VA-R		LA-R	
n	LVs	Loadings	LVs	Loadings	LVs	Loadings	LVs	Loadings
50	0.323	0.073	0.382	0.160	0.323	0.073	0.474	0.134
120	0.268	0.037	0.300	0.125	0.269	0.037	0.337	0.085
190	0.246	0.026	0.266	0.105	0.247	0.027	0.312	0.068
260	0.238	0.021	0.252	0.082	0.238	0.021	0.302	0.063

The Indonesian birds data: Bernoulli GLLVM

Table S2.10. Average biases, root mean squared errors (RMSEs), coverage probabilities of 95% confidence intervals and mean confidence interval widths (CI) for GLLVM estimates based on variational approximation and Laplace approximation methods. The true model parameters were obtained by fitting a Bernoulli GLLVM with random row effects and probit link function fitted for the Indonesian birds data with presence-absences of $m = 30, 60, 100$ and 140 species recorded at $n = 37$ sites.

		VA-`TMB`				LA-`TMB`			
m		Bias	RMSE	Cover	CI	Bias	RMSE	Cover	CI
30	β_0	0.12	0.33	0.93	1.29	-5.71	21.40	0.79	6.84
	$\log\sigma$	-2.90	13.98	0.99	19.66	-0.01	0.04	0.90	0.71
60	β_0	-0.01	0.32	0.97	1.50	-1.05	12.54	0.86	3.23
	$\log\sigma$	-0.17	0.45	0.94	0.96	0.04	0.02	0.84	0.57
100	β_0	-0.03	0.35	0.97	1.55	-0.07	3.29	0.90	1.89
	$\log\sigma$	-0.00	0.02	0.92	0.55	0.04	0.02	0.88	0.52
140	β_0	-0.03	0.37	0.97	1.57	-0.05	0.75	0.92	1.72
	$\log\sigma$	0.02	0.02	0.93	0.52	0.04	0.02	0.89	0.51

Table S2.11. Median VE values of Bernoulli GLLVMs for 500 simulated datasets. Models were fitted using the variational approximation and the Laplace approximation methods. The datasets were based on a Bernoulli GLLVM with probit link function and random row effects fitted for the Indonesian birds data with presence-absences of $m = 30, 60, 100$ and 140 species recorded at $n = 37$ sites.

m	VA-`TMB`	LA-`TMB`
30	0.22	0.14
60	0.39	0.31
100	0.44	0.39
140	0.47	0.44

Table S2.12. Scaled mean Procrustes errors of predicted latent variables and estimated latent variable loadings for GLLVM estimates based on variational approximation and Laplace approximation methods. Values are scaled with the number of sites and number of species for comparisons. The true model parameters were obtained by fitting a Bernoulli GLLVM with random row effect and probit link function for Indonesian birds data with presence-absences of $m = 30, 60, 100$ and 140 species recorded at $n = 37$ sites.

	VA		LA	
m	LVs	Loadings	LVs	Loadings
30	0.569	0.112	0.576	0.140
60	0.271	0.096	0.306	0.149
100	0.175	0.091	0.199	0.125
140	0.113	0.090	0.125	0.128
