# Supplementary material for: Efficient estimation of generalized linear latent variable models
Source: PLoS One. 2019 May 1;14(5):e0216129. doi: 10.1371/journal.pone.0216129 (PMC6493759; doi:10.1371/journal.pone.0216129)
Supplement: S3 Appendix — (PDF) [file pone.0216129.s003.pdf]

S3 Appendix. Starting value comparisons

Table S3.1. Average biases, root mean squared errors (RMSEs), coverage probabilities of 95% confidence intervals and mean confidence intervals widths (CIw) for β_0 estimates as well as mean procrustes errors of predicted latent variables and estimated latent variable loadings based on the plain `R` and the `TMB` implementations for the variational approximation and the Laplace approximation methods, with four starting value methods `res3`, `res`, `zero` and `random`. The true model parameters were obtained by fitting a Bernoulli GLLVM with probit link function for the Indonesian birds data with presence-absences of $m = 30, 60, 100$ and 140 species recorded at $n = 37$ sites.

		VA-TMB						VA-R					
m		Bias	RMSE	Cover	CIw	LVs	Loadings	Bias	RMSE	Cover	CIw	LVs	Loadings
30	`res3`	0.048	0.299	0.934	1.272	0.557	0.098	0.048	0.300	0.933	1.269	0.559	0.123
	`res`	0.048	0.299	0.934	1.272	0.556	0.098	0.048	0.299	0.933	1.269	0.554	0.122
	`zero`	0.038	0.329	0.933	1.271	0.558	0.123	0.035	0.319	0.934	1.269	0.556	0.122
	`random`	0.054	0.299	0.930	1.268	0.558	0.098	0.056	0.308	0.923	1.263	0.559	0.123
60	`res3`	-0.028	0.314	0.976	1.545	0.185	0.098	-0.029	0.315	0.976	1.542	0.185	0.098
	`res`	-0.028	0.314	0.976	1.545	0.185	0.098	-0.029	0.315	0.976	1.541	0.185	0.098
	`zero`	-0.028	0.318	0.975	1.542	0.185	0.098	-0.029	0.316	0.976	1.541	0.185	0.098
	`random`	-0.023	0.310	0.975	1.540	0.185	0.098	-0.024	0.318	0.968	1.528	0.186	0.099
100	`res3`	-0.031	0.352	0.961	1.553	0.129	0.095	-0.031	0.352	0.961	1.551	0.129	0.096
	`res`	-0.031	0.352	0.961	1.553	0.129	0.095	-0.031	0.351	0.961	1.551	0.128	0.095
	`zero`	-0.034	0.348	0.969	1.579	0.129	0.095	-0.044	0.344	0.978	1.614	0.128	0.095
	`random`	-0.032	0.352	0.966	1.576	0.129	0.096	-0.030	0.357	0.962	1.553	0.129	0.096
140	`res3`	-0.028	0.388	0.958	1.569	0.109	0.113	-0.028	0.388	0.958	1.561	0.112	0.131
	`res`	-0.028	0.388	0.958	1.569	0.121	-0.101	-0.028	0.388	0.958	1.562	0.131	0.126
	`zero`	-0.037	0.381	0.967	1.606	0.137	-0.089	-0.048	0.366	0.979	1.661	0.492	0.158
	`random`	-0.029	0.387	0.960	1.583	0.110	0.130	-0.025	0.393	0.955	1.545	0.496	0.222
		LA-TMB						LA-R					
30	`res3`	-6.021	22.506	0.693	4.070	0.628	0.136	0.091	0.494	0.780	1.139	0.600	0.153
	`res`	-5.288	20.785	0.727	4.711	0.615	0.139	-0.012	0.485	0.807	1.268	0.667	0.173
	`zero`	-4.755	18.257	0.733	4.663	0.626	0.155	-0.062	0.504	0.839	1.372	0.746	0.196
	`random`	-5.260	21.121	0.733	5.019	0.615	0.202	0.023	0.559	0.785	1.306	0.676	0.174
60	`res3`	-0.724	17.415	0.876	4.847	0.206	0.158	-0.021	0.562	0.808	1.258	0.205	0.134
	`res`	-0.536	13.862	0.893	5.234	0.204	0.160	-0.151	0.762	0.830	1.572	0.291	0.141
	`zero`	-0.499	13.525	0.889	4.873	0.206	0.160	-0.145	0.696	0.861	1.562	0.503	0.210
	`random`	-0.560	14.231	0.893	5.420	0.207	0.147	0.046	0.690	0.751	1.416	0.365	0.193
100	`res3`	-0.056	6.748	0.902	2.965	0.145	0.129	-0.021	0.534	0.832	1.211	0.148	0.136
	`res`	-0.054	5.372	0.915	3.186	0.144	0.130	-0.123	0.950	0.836	1.687	0.176	0.139
	`zero`	-0.054	5.542	0.914	3.035	0.145	0.150	-0.109	0.779	0.858	1.602	0.521	0.180
	`random`	-0.056	5.047	0.917	3.361	0.146	0.135	0.097	0.700	0.715	1.381	0.392	0.200
140	`res3`	-0.037	1.258	0.907	1.734	0.109	0.113	-0.030	0.595	0.828	1.158	0.112	0.131
	`res`	-0.037	1.045	0.918	1.767	0.109	0.121	-0.101	0.936	0.828	1.420	0.131	0.126
	`zero`	-0.037	2.291	0.920	1.785	0.108	0.137	-0.091	0.810	0.845	1.388	0.492	0.158
	`random`	-0.036	1.091	0.919	1.840	0.110	0.130	0.170	0.642	0.634	1.107	0.496	0.222

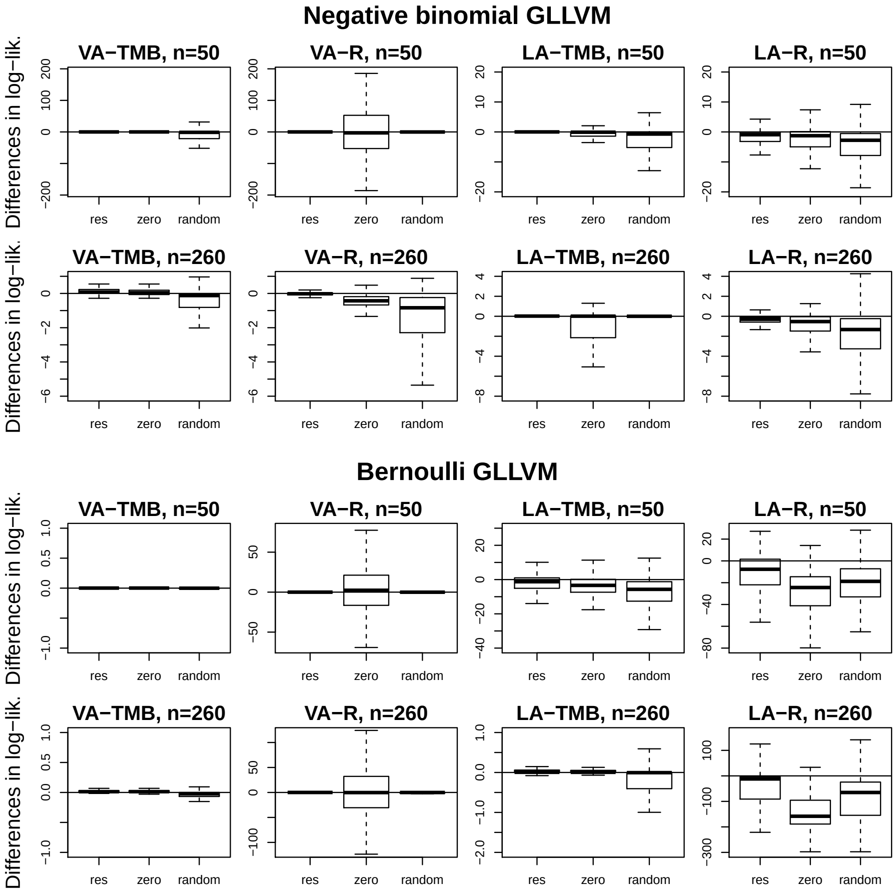

Fig S3.1. Differences in log-likelihood value when strategies `res`, `zero` and `random` are compared to `res3`. The true models were based on negative binomial GLLVMs (top) and Bernoulli GLLVMs (bottom) fitted for the testate amoebae data with $m = 48$ species recorded at $n = 50$ and $n = 260$ sites. Negative value means that performance of the corresponding starting value strategy is worse than that of `res3`. Notice that each column has its own scale.

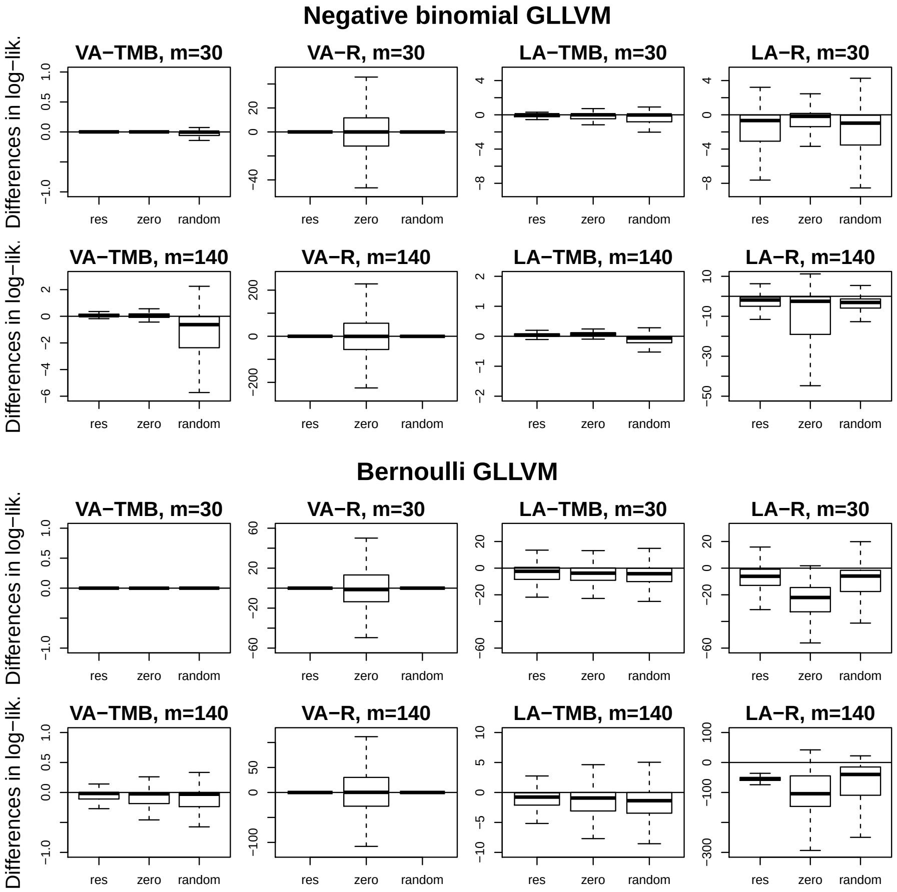

Fig S3.2. Differences in log-likelihood value when strategies `res`, `zero` and `random` are compared to `res3`. The true models were based on negative binomial GLLVMs (top) and Bernoulli GLLVMs (bottom) fitted for the Indonesian bird data with $m = 30$ and $m = 140$ species recorded at $n = 37$ sites. Negative value means that performance of the corresponding starting value strategy is worse than that of `res3`. Notice that each column has its own scale.
